# Deprescribing in cardiometabolic conditions in older patients: a systematic review

**DOI:** 10.1007/s11357-023-00852-z

**Published:** 2023-07-05

**Authors:** Elizabeth Hickman, Mansha Seawoodharry, Clare Gillies, Kamlesh Khunti, Samuel Seidu

**Affiliations:** Diabetes Research Centre, Leicester Diabetes Centre, University of Leicester, Leicester General Hospital, Gwendolen Road, Leicester, LE5 4WP UK

**Keywords:** Deprescribing, End-of-life, Cardiometabolic, Multimorbidity, Polypharmacy

## Abstract

We conduct a systematic review to investigate current deprescribing practices and evaluate outcomes and adverse events with deprescribing of preventive medications in older patients with either an end-of-life designation or residing in long-term care facilities with cardiometabolic conditions. Studies were identified using a literature search of MEDLINE, EMBASE, Web of Science, clinicaltrials.gov.uk, CINAHLS, and the Cochrane Register from inception to March 2022. Studies reviewed included observational studies and randomised control trials (RCTs). Data was extracted on baseline characteristics, deprescribing rates, adverse events and outcomes, and quality of life indicators, and was discussed using a narrative approach. Thirteen studies were identified for inclusion. Deprescribing approaches included complete withdrawal, dose reduction or tapering, or switching to an alternative medication, for at least one preventive medication. Deprescribing success rates ranged from 27 to 94.7%. The studies reported no significant changes in laboratory values or adverse outcomes but did find mixed outcomes for hospitalisations and a slight increase in mortality rates when comparing the intervention and control groups. Lack of good-quality randomised control trials suggests that deprescribing in the older population residing in long-term care facilities with cardiometabolic conditions and multimorbidity is feasible when controlled and regularly monitored by an appropriate healthcare clinician, and that the benefits outweigh the potential harm in this cohort of patients. Due to the limited evidence and the heterogeneity of studies, a meta-analysis was not performed and as such further research is required to assess the benefits of deprescribing in this patient population. Systematic review registration: PROSPERO CRD42021291061.

**Supplementary information:**

The online version contains supplementary material available at 10.1007/s11357-023-00852-z.

## Introduction

Cardiometabolic conditions are a group of commonly known interrelated conditions and risk factors that include diabetes, strokes, cardiovascular diseases, chronic kidney disease, hypertension, dyslipidaemia, and non-alcoholic fatty liver disease and are the world’s leading cause of mortality according to WHO 2022 [1]. There is a strong relation between diabetes and cardiovascular health which provides a compelling and justifiable cause to assess the impacts of deprescribing in this population as most patients progress from having a single disease to multimorbidity contributing to the high mortality rates seen worldwide [2].

Frail, multimorbid patients with cardiometabolic conditions are disproportionately affected by pill-burden, mismanagement, and inappropriate target goals and treatment outcomes, often due to a lack of evidence base and guidelines to aid clinicians to safely and effectively manage them. The focus of management for frail and older patients should be omitting medications prescribed earlier which prevent long-term complications, since their life expectancy is now markedly reduced, and they are unlikely to live long enough to develop micro- and macro-vascular complications and continuing the medications may in fact cause harm. Focusing on improving the quality of patients’ lives through the use of medications with minimal or no side effects yet are effective enough to prevent acute symptoms or complications should be encouraged.

In older patients living with frailty and multimorbidity and approaching the end-of-life, attempting to manage them with current guidelines to achieve target goals such as glycaemic control, systolic blood pressure, and lipid levels becomes more complex as the line between maintaining therapeutic targets and ensuring the quality of patients’ lives is upheld becomes increasingly more blurred. Increased incidences of adverse effects such as hypoglycaemia due to aggressive therapeutic control, along with an increased prevalence of hospitalisations due to overtreatment of conditions, leads to new guidance in this field being of vital importance to maintain patient safety [3]. With the limited involvement of older people within randomised controlled trials (RCTs), heterogeneity in medication safety is unknown and further research into effective and safe prescribing techniques, commonly being termed as “deprescribing”, are required.

Intensive control of targets such as blood sugars, systolic blood pressure, and lipid levels in a patient’s early years has been commonly known to reduce the long-term impacts of their associated diseases; however, there are considerable gaps in the current knowledge into the mechanisms responsible and the ideal targets for patients living with frailty and multimorbidity [4]. In older patients, target setting should be individualised to consider the needs of the presenting individual, with the mainstay of treatment being to preserve quality of life (QoL), with the prevention of long-term complications being deemed unnecessary. Individualising targets can capture the target goals from the individual’s perspective that is often lost when using guideline set targets, such as NICE guidelines [NG28] or [NG136], which motivate current clinician decisions and thereby allow for a patient-centred approach. Targets such as HbA1c, systolic blood pressure, low-density lipoproteins (LDL), and high-density lipoproteins (HDL) levels, to name a few, should be reduced and consideration into minimising adverse effects of associated treatments should be highlighted, whilst avoiding acute complications such as metabolic decompensation. Recommended targets should focus on maintaining HbA1c levels below 8.5% (< 69 mmol/l), with fasting plasma glucose of between 7.0 and 10.0 mmol, whilst blood pressure readings should be increased to a new goal of < 150/90 [4]. In patients with minimal life expectancy, cardio-protective medications such as beta-blockers, ACE inhibitors, aspirin, and statins could be safely stopped. Those on combination oral diabetes medications could be converted onto insulin alone either BD (“*bis in die*” meaning “*twice daily*”) or OD (“*omne in die*” meaning “*once a day*”), continually relaxing therapeutic targets as end-of-life is approached, whilst continually assessing for symptoms of hypo- (< 4 mmol/l) and hyper- (> 15 mmol/l) glycaemia [5].

### Deprescribing and deprescribing tools

Deprescribing is a systematic process which can safely reduce medications in clinical practice through deintensification whereby they are simplified, reduced, or completely withdrawn in an effort to prevent the risk of polypharmacy [3], with many deprescribing tools having been recently highlighted in aiding clinicians make these deprescribing decisions including the STOPP/START tool, the Beers criteria, the IMPACT tool, the 7-steps approach, and the NO TEARS guideline [4]. Deintensification of medications is considered to be part of safe prescribing practices and focuses on the benefits and harms of medications, along with assessing the long-term micro- and macro-vascular complications. Deprescribing should be undertaken by an appropriate healthcare clinician, such as the GP or a prescribing pharmacist, with the focus of discussions identifying patient preferences, life expectancy, drug-burden, and goals of care. Whilst deprescribing tools have been identified to help clinicians, it is important to recognise deprescribing as a partnership between the patient and the healthcare clinician [6].

### Criteria, rationale, and ethical basis for deprescribing

Literature highlighting healthcare professionals’ attitudes towards deprescribing has identified that many guidelines for use with patients with a cardiometabolic condition has completely omitted or not addressed the management of those with frailty, multimorbidity, or an end-of-life status. Patients with these additional needs increase the complexity of disease-based care and ultimately render the guideline or evidence-base from which they are compiled unsafe for this patient cohort. Clinicians have raised concerns for the safety of these patients and have admitted to feeling incompetent when managing these patients in both primary and secondary care [7].

Deciding the point upon which a patient is approaching the end-of-life has been a topical discussion for many years and has to be decided upon on an individual basis. There are a number of instances when a reduction in medication may be identified as being appropriate, such instances include reducing medications at the point whereby the prevention of long-term micro- and macro-vascular complications is deemed to be no longer necessary, the occurrence of an adverse drug reaction (ADE), when new individualised targets have been set for the patient, when polypharmacy or a prescribing cascade is identified (i.e., when a patient is taking > 5 medications for long-term purposes), and at the end of life as part of palliative care. The above outlined deprescribing tools can aid clinicians in deciding appropriate timelines to begin deprescribing initiatives, but identifying new individualised targets and potential contraindications and adverse side effects of medications should also be of use.

When considering patients with diabetes, as an example, there are a few ways in which the highlighted triggers might prompt clinicians to initiate deprescribing processes. Metformin might be stopped in those who have a creatinine level above 150 mmol/l or an eGFR < 30 or when its effect on the gastrointestinal system becomes increasingly more troublesome. A reduced renal clearance can result in the formation of lactic acidosis with metformin use, an increased risk of hypoglycaemia with nepaglinide and sulphonylureas and oedema with glitazone use [4]. Pioglitazone should be avoided in those with heart failure whilst sulphonylureas should be stopped in people with very low renal function to reduce the risk of hypoglycaemia. Incretin-based therapies should be tapered in line with decreasing renal function as suggested in the respective summary product characteristics and glucagon-like peptide-1 receptor agonists (GLP-1 analogues) specifically should be withdrawn in those experiencing significant gastrointestinal side effects [5].

Current guidelines outlining the management of cardiometabolic conditions do not identify how long associated medications should be administered for and are therefore usually administered over many years. With the long-term benefits and risks of diabetes and cardiovascular medications being unknown in frail adults living with multimorbidity, medications need to be individualised in accordance with their life expectancy, treatment outcomes, individualised targets, and patient preferences [8].

The Four Principles of ethics as outlined by Beauchamp and Childress 2012, namely, beneficence, non-maleficence, justice, and autonomy, should guide clinicians when identifying patients to begin deintensification of medications [9]. Informed consent is the process by which information is efficiently presented to the patient and time allowed for the patient to reflect on the information provided. It also implies that the consent can be withdrawn at any time. These principles provide a good pragmatic framework for clinicians to apply to patients and make decisions that are morally acceptable to both the clinician and the patient. Anderson et al. described medication deprescribing as an “active medical decision” influenced by barriers and enablers [10]. Guidelines need to decide whether deprescribing is seen as an *action* or the *discontinuation of a previous action*, which makes the consideration of beneficence and non-maleficence difficult to contemplate.

Justice refers to not only the appropriate use of resources but also the right to equal treatments [11]. With ageing, there becomes a shift between the benefits and the risks of the medication being used, and as such it is important to recognise that deprescribing is not the omission of a medication but the continuous reflection upon a patient’s management and treatment goals [11]. When considering deprescribing interventions, it is of high clinical importance that the clinician is aware of the patient’s level of capacity, their beliefs, and preferences, as well as their current clinical state.

### Legal aspects of deprescribing

With emerging evidence supporting the necessity of deprescribing, many clinicians are concerned with the possibility of litigation. One of the key aspects of tort law is to protect patients by deterring clinicians from providing substandard clinical care which has the ability to cause harm to the patient [9]. Some of the possible contributors to tort law cases associated with deprescribing include clinical negligence and not providing appropriate informed consent.

Literature has predicted that deprescribing malpractice claims have the potential to be split into two categories, (1) a failure to deprescribe when it should be deemed necessary or (2) deprescribing in a situation whereby it was necessary to continue a medication. These cases could only raise litigation issues should harm come to a patient. Informed consent may be implicitly needed in situations where there is a lack of evidence-base, clinical guidelines, or trust-based policies, whereby shared decision-making tools and aids should be utilised to maintain patient-centred care.

Legally, deprescribing is no different to prescribing. Safe prescribing practices whether that be prescribing or deprescribing require the ongoing monitoring of patients, with informed consent being obtained by the patient [12]. When deprescribing is performed in partnership with the patient and/or caregiver, supported by the appropriate knowledge and skills, and considering the values and beliefs of each patient, the law presents no barriers to deprescribing practices, with its initiatives lying concordant with the ethical principles of Beauchamp and Champ [13, 14].

As this is a rapidly developing field, this systematic review aims to provide a review of the evidence for deprescribing in the older population with a focus on those residing in long-term care facilities with cardiometabolic conditions and multimorbidity, nearing their end of life. This will aid clinicians who focus on the management of patients with cardiometabolic conditions in the deprescribing process by providing a review of current literature, assessing the safety and efficacy of previous deprescribing interventions and to assess the adverse outcomes, associated laboratory values, hospitalisations, and mortality rates deduced from the included studies. Highlighting the importance of deprescribing should improve collaborative discussions surrounding appropriate medication prescribing with the aim of integrating the technique into routine management for these patients.

## Methods

This review is reported in accordance with PRISMA [15] and MOOSE [16] guidelines, with the corresponding checklists provided in Supplementary Material Tables S1 and S2 respectively. Predefined criteria (Supplementary Table S3) were used to assess eligibility of studies for inclusion within the review, which was registered with the prospective register of systematic reviews (PROSPERO) as CRD42021291061.

### Definition of terms

The population, intervention, comparator, and outcome (PICO) framework was used to identify included studies (Supplementary Table S3) containing patients aged 65 years or more with a cardiometabolic condition residing in a long-term care facility, including nursing homes, care homes, hospices, and residential homes, or who had an end-of-life designation, who were taking medications for the therapeutic intervention of their conditions. The intervention was the deprescribing approaches defined as either the complete withdrawal, reduction in dose or regime, tapering of a medication, or switching to a more alternative medication. The control was patients who did not experience deprescribing approaches and had their usual care continued. Outcomes of interest included laboratory values, hospitalisations, GP visits, complications, adverse events, falls, syncope events, mortality, quality of life indicators, and patient satisfaction scores.

### Search strategy

Databases searched were MEDLINE, EMBASE, Web of Science, CINAHL, clinicaltrials.gov.uk, and the Cochrane Central Register of Clinical Trials from inception to March 2022. Keyword searches and MeSH searches were used to identify papers of interest. These terms were related to deprescribing (e.g., “deprescribe”, “deintensification”, “discontinue”, “cessation”), diabetes (e.g., “T2DM”, “type 2 diabetes”, “diabetes mellitus”), medication (e.g., “prescription”, “antihyperglycaemic”, “antihypertensive”, “statins”), palliative (e.g., “palliation”, “terminal”, “end-of-life”), cardiometabolic (e.g., “stroke”, “transient ischaemic attack”, “hypercholesterolaemia”, “heart attack”), and elderly (e.g., “aged”, “elder”, “geriatric”). The language was restricted to English language only due to translational services being unavailable. Citation searches were carried out on all papers of “exceptional interest”; these were separately acknowledged and are outlined on the PRISMA flowchart (Appendix Fig. 1). All duplicate records were removed from the compilation of results. The full systematic search strategy can be found in Supplementary Material Figure S1.

### Eligibility criteria

We searched for randomised controlled trials (RCTs) inclusive of cluster and pragmatic trials, retrospective and prospective cohort studies, and pre-post studies, which identified (i) older patients aged 65 years or more with cardiometabolic conditions such as type 2 diabetes, stroke or transient ischaemic attack (TIA), hypertension, cardiovascular diseases, hypercholesterolaemia, and chronic kidney disease (CKD) who were taking preventive medications for their condition; (ii) reported deprescribing approaches (complete withdrawal of a medication, reducing a dose or regime, tapering of a medications, or switching to an alternative medication); (iii) reported outcomes including laboratory values, hospitalisations, GP visits, mortality rates, quality of life indicators, complications, patient satisfaction, and deprescribing success rate; and (iv) patients residing in a long-term care facility or had an end-of-life designation. Studies were included if the study participants were aged 65 years or more, or the mean age of the study participants was 65 years or more or the data for the over 65 cohort could be identified from the study data.

Studies were excluded from the review if (i) no deprescribing interventions were reported, (ii) study participants did not have a diagnosis of a cardiometabolic condition, (iii) there were no reported outcomes, and (iv) studies that did not include patients residing in long-term care facilities or those without an end-of-life designation.

### Data extraction and risk of bias assessment

Two authors (EH and MS) independently screened the full titles and abstracts against the pre-defined inclusion criteria, and then went on to independently review the full-text articles for inclusion, whilst one author independently extracted the data (EH) and a second author reviewed the extracted data (MS) with that found in the original articles using the standardised data extraction form. Papers were screened and data collected by two independent authors, with a third author available to settle any raised disputes.

Extracted data included geographical location of study, publication date, study design, study setting, mean age, baseline characteristics, sample size, percentage of females, duration of follow-up, medications deprescribed, deprescribing intervention, deprescribing success rate, comorbidities, medical interventions, adverse outcomes, mortality, complications, quality of life indicators, and patient satisfaction scores. Any disagreements that arose were mediated by a third author (CG) and agreement reached by consensus.

The included studies were independently assessed for risk of bias using the Newcastle-Ottawa scale for randomised controlled trials and cohort studies, and the NIHR 12-question risk of bias tool for pre-post studies. The validated tools assess the selection of participants scoring out of 4 stars, comparability scoring out of two stars, and the ascertainment of outcomes of interest scoring out of three stars, totalling a maximum score of 9 stars. A study that obtains the maximum score of 9 stars is identified as being of the highest study quality. The NIH risk of bias tool scores a maximum of 12 points, with a study scoring 12 points being identified as the highest study quality [17]. Due to the heterogeneity of the included studies, we were unable to conduct a meta-analysis on the studies, and therefore the results have been narratively synthesised.

## Results

### Study identification and selection

The PRISMA flow diagram shows the identification of eligible studies through to the final included studies within the review. The literature searching of databases yielded 7402 eligible papers which were independently screened by two authors against the pre-defined eligibility criteria. After initial screening based upon full titles and abstracts, 62 papers were identified as being eligible for inclusion. Following full text retrieval and analysis, 10 papers were included within the review, and 3 additional papers were identified from citation searches with 56 papers excluded due to being (i) conference abstracts (*n* = 8), (ii) systematic reviews (*n* = 5), (iii) wrong study population (*n* = 3), (iv) wrong medication (*n* = 2), (v) placebo studies (*n* = 3), (vi) no deprescribing interventions were noted (*n* = 29), (vii) written in a language other than English (*n* = 1), and (vii) clinical trials who had not yet begun recruiting study participants (*n* = 5).

### Study characteristics

Of the included studies (Appendix Tables 1 and 2), the majority were conducted in the USA (*n* = 8) and the rest included Europe (*n* = 3), Malaysia (*n* = 1), and Canada (*n* = 1). One of the studies carried out in Europe also included sites across Israel. Eleven of the studies were conducted on patients residing in nursing homes, one of the studies was conducted on patients with life-limiting conditions and one study was conducted across geriatric institutions. The age range of patients spanned from 65 to 101 years, with the mean study age of participants across all studies ranging from 74.1 to 87.2 years. The types of study design included retrospective cohorts (*n* = 5), prospective cohorts (*n* = 2), pre-post studies (*n* = 1), cluster randomised control trials (*n* = 1), pragmatic clinical trials (*n* = 1), double-blind clinical trial (*n* = 1), and longitudinal multi-centre cohorts (*n* = 2). The sample size of included participants ranged from 14 to 13,844. The duration of study follow-up ranged from 30 days to 12 months, with the majority of the studies having a follow-up duration of 3 months (*n* = 4) or 12 months (*n* = 3).

### Study quality

The cohort studies were assessed as being of good quality ranging from 5 to 7 stars, the RCTs were assessed as being of good quality ranging from 5 to 6 stars, and the pre-post study was also assessed as being of good quality scoring 7 stars. No study was identified as being of the highest quality scoring either a 9 (for the NOS) or 12 (for the NIH bias tool) (Supplementary Tables S4, S5, and S6 respectively) [18].

### Baseline results

The 13 papers included studies involving the discontinuation of statins [19, 20], antihypertensives [21–23], anti-diabetics [24, 25], aspirin [26], digoxin [27, 28] , diuretics [29], and a combination of multiple medications [30, 31] (Supplementary Table 1.4). All of the included studies presented deprescribing as complete withdrawal or stopping of a medication (*n* = 13); however, other types of deprescribing were reported including dose reduction (*n* = 1). Switching to an alternative medication was not reported in any of the included studies. The main considerations for deintensification of medications included patients being at the end of their lives with an ideal of improving the quality of patients’ lives, the risks of medications now outweighing the benefits due to increased frailty, comorbidities and end-of-life status, and no current clinical indication for the medication. Two studies included the deprescription of multiple medications but were included in this review as the majority of medications deprescribed were for the intervention of cardiometabolic conditions. One study described the deprescribing of potentially inappropriate medications (PIMs) in nursing home residents using the Screening Tool of Older Persons Prescriptions (STOPP) criteria; whilst not directly related to the review question, it was included due to its high relevance to the topic. A large portion of the studies were before and after studies, six studies compared deprescribing approaches to a control group, five received usual care, and one received a placebo.

### Deprescribing approaches

Across the 13 papers, the deprescribing success rates ranged from 27.0 to 94.7%. The discontinuation of anti-diabetics found a deprescribing success rate of 45.5% (*n* = 1390) in a study among 3056 Veterans in an end-of-life VA nursing home in the USA, where anti-diabetics were discontinued or dose reduced for a consecutive 7-day period in those who had HbA1c < 7.0% [24] and 75.0% (*n* = 24) in a study of 98 patients across Swedish nursing homes, where patients insulin doses were reduced or completely withdrawn, or there was a reduction in either the dosage or the number of oral antihyperglycaemic agents, in comparison with a control cohort where no alterations were made to diabetes medications [24]. The cumulative incidence of deprescribing at 15, 30, 60, and 90 days was 22.0%, 30.1%, 38.0%, and 45.5%, respectively [24].

In a study by Gulla et al., 51.2% (*n* = 151) of patients were successfully withdrawn from their antihypertensive treatment in 295 patients aged > 65 years old with multimorbidity in nursing homes in Norway. In a study carried out by Song et al., across long stay nursing home residents in the USA found 2212 residents who were being over-aggressively treated for their hypertension. 11.0% (*n* = 243) experienced deprescribing interventions due to fitting into one of the following categories: (1) low blood pressure, (2) > 1 antihypertensive drug, (3) no CHF, (4) fracture from index fall, (5) older age. A study carried out by Vu et al., amongst 10,574 older Veterans with a limited life expectancy (LLE) or advance directive (AD) and a diagnosis of hypertension receiving at least one antihypertensive medication and a mean daily systolic blood pressure (SBP) of < 120 mmHg, determined a deprescribing success rate of 42.0% (95% CI 40 to 42%) (*n* = 4335) following discontinuation after 30 days. Out of the study participants, 39.4% had > 5 comorbidities related to cardiovascular disease (CVD) risk including hyperlipidaemia (64.2%), congestive heart failure (CHF) (50.9%), diabetes (47.4%), AF (22.1%), renal failure (16.8%), and a recent stroke or transient ischaemic attack (TIA) (16.5%), with 60.5% taking multiple antihypertensives. The most commonly used antihypertensives included beta-blockers (66.9%), loop diuretics (38.3%), and alpha-1 blockers (37.3%), reiterating the high number of patients with CVD in the cohort. Deprescription of antihypertensive medications did not appear to significantly increase the SBP in these cohorts of patients. Adverse events, such as falls, a decline in cognitive function, cardiovascular events, or a decline in general daily functioning, were not noted as being of great significance in the sampled populations. Gulla et al. found that in the initial stages of antihypertensive deprescribing, there was a lower rate of hospitalisation in the intervention arm (5%, *n* = 7) compared with the control arm (13%, *n* = 14); however, the rate of those hospitalised was increased during the latter stages of deprescribing in the intervention arm (6%, *n* = 7) compared with the control arm (13%, *n* = 17). Mortality was found to be marginally higher in the intervention arm (12%, *n* = 17) compared to the control arm (11%, *n* = 12). Vu et al. did not discuss mortality rates within their study for us to compare results.

In a retrospective cohort study performed by Thorpe et al., across 13,110 Veterans with coronary artery disease (CAD), stroke, or diabetes with a limited life expectancy receiving statins in nursing homes, a deprescribing success rate was deduced to be 31.0% (95% CI 30.0 to 32.0%) (*n* = 4064) following 90 days of discontinuation. Discontinuation of statins was determined to be greater in those with an end-of-life (EoL) designation at 52.0% (95% CI 50.0 to 55.0%) (*n* = 2113) compared to 25.0% (95% CI 24.0 to 26.0%) (*n* = 1016) without end-of life designation. A block randomise trial by Kutner et al., focusing on the discontinuation of statins for 12 months, found an overall improvement of quality of life (QoL) indicators in the intervention arm of 7.11 compared with the control arm 6.85 using the McGill QoL Scoring Tool, whilst 13 CVD events were noted in the intervention arm and 11 in the control arm. Mortality was deduced to be 23.8% (*n* = 45) compared with 20.3% (*n* = 39) in the intervention arm compared with the control arm respectively. Statin discontinuation was noted to be safe with no increases in mortality noted by Thorpe et al., as no control group was present, whilst Kutner et al. found a slight increase in mortality rates; however, no clinical significance was noted. Secondary benefits were noted to improve in the sampled cohorts including a reduction in pill-burden (*p* = 0.03), improved quality of life, and a decrease in associated medication costs. Quality of life markers, determined using the McGill QoL scoring tool, was improved with a total McGill score of *p* = 0.04. Total cost saved by discontinuing medications in the sampled population was determined to be $3.37 per day (95% CI: 2.83 to 3.91). Mortality was determined to be 23% (*n* = 3015), with 57% (*n* = 1719) of those who passed having a pre-designated EoL status by Thorpe et al., whilst Kutner et al. found that mortality was slightly increased in the deprescribing arm compared with the control arm.

In a double-blind randomised control trial undertaken by Myers et al., in 77 older patients across two geriatric institutions using diuretics not concomitantly with digoxin for CHF or pulmonary hypertension, where participants were randomly allocated to either continue diuretic therapy (*n* = 39) or given a placebo (*n* = 38) found a 74.4% (*n* = 28) deprescribing success rate after 12 months of follow-up. The mean blood pressure of the placebo subjects at 12 months (*n* = 26) was (SBP/DBP) 135 ± 4.6 / 74.3 ± 2.9 mmHg which was moderately higher than at baseline 129.3 ± 4.1 / 73.6 ± 2.5 mmHg; however, there was no difference noted between the SBP and DBP for both the control arm and the intervention arm including supine, sitting, and erect, with a decreased change in SBP and DBP for postural hypotension in the intervention arm. Heart rates were found to the consistent across both arms of the study. No major cardiovascular events were noted when deprescription occurred; however, 15.38% (*n* = 6) in the intervention arm and 5.26% (*n* = 2) in the control arm developed CHF, whilst 5.65% (*n* = 2) in the placebo group developed a noticeable increase in overall SBP and DBP values.

In a retrospective cohort study carried out by Springer et al., across 13,844 Veterans aged > 65 years with limited life expectancy with a history of CAD, stroke, or TIA, a deprescribing success rate for aspirin was found to be 27.0% (*n* = 3738) (95% CI 26.0 to 28.0%), split across 34.0% (*n* = 1270) (95% CI 33.0 to 36.0%) with explicit limited prognosis documentation, and 24.0% (*n* = 897) (95% CI 23.0 to 25.0%) without [26]. The whole study sample had limited prognosis documentation; however, not all participants had the correct limited prognosis documentation contained within their medical records. Discontinuation of aspirin was higher amongst those with renal failure and lower amongst those with diabetes and congestive heart failure. Mortality rates and adverse events were not sampled and recorded for the study; however, it was of note that there was a 41.66% increase of deprescribing initiatives being undertaken in those with an explicit EoL designation allowing us to infer that EoL status heavily impacts a clinician’s likelihood to deprescribe aspirin in older patients.

In a pre-post study undertaken by Forman et al., amongst 14 study participants across two urban nursing homes, a deprescribing success rate of 92.8% (*n* = 13) was determined for the cardiac glycoside digoxin, with patients having their digoxin deprescribed when found to be in normal sinus rhythm (NSR) or normal echocardiogram left ventricular (LV) ejection fraction (EF) [27]. A study carried out by Wilkins et al., in nursing home residents with no evidence of CHF, found a deprescribing success rate of 94.7% (*n* = 18), with 81.3% showing no changes in clinical status [28]. Three adverse events were highlighted across the studies, with Forman et al. finding one patient had a drop in left ventricular ejection fraction rate from 60 to 50%; however, no participants showed signs of clinical deterioration, such as an episode of CHF or dysrhythmia, whilst Wilkins et al. had one participant who developed atrial fibrillation (AF) with a ventricular rate of 80–90 bpm (beats per minute) on day 5 which spontaneously resolved and reverted back to NSR for the remainder of the study, and one participant who developed dyspnoea on day 10. Deprescription of digoxin was found to increase EF in 28.6% (*n* = 4) of participants for Forman et al., whilst Wilkins et al. had to restart digoxin in one patient with sinus tachycardia and furosemide in another patient who had an episode on CHF. Overall, the studies highlighted that there were minimal impacts to decreasing digoxin use in this cohort of patients, with Wilkins et al. finding that there was a mortality rate of 0% and Forman et al. did not report upon mortality rates, highlighting that digoxin can be safely reduced or stopped in the older patient.

Al Aqqad et al. carried out a study on 211 patients aged 65 years in four non-governmental nursing homes in Malaysia taking at least 1 medication and had PIMs stopped according to the STOPP criteria which were reassessed after 3 months. The mean number of medications prescribed was 4, with a range of 1 to 14. There was a 5.1% reduction in PIMs across the 3 months, with a total prevalence of 23.7% at baseline, reducing to 18.6% at follow-up. In a study by Onder et al., across 1843 nursing home residents aged > 65 years taking more than 5 medications across Europe (Czech Republic, England, Finland, France, Germany, Italy, and the Netherlands) and Israel showed a deprescribing rate of 35.7% (*n* = 658). The mean number of medications per resident was determined to be 8.6 at baseline. The mean number of medications in the control arm increased from a baseline of 8.6 ± 2.9 to 9.7 ± 2.9 at 12 months, whilst in the deprescribing arm the mean number of medications at baseline was 9.0 ± 2.8 decreasing to 6.8 ± 3.1 at 12 months. Medications commonly deprescribed were antiplatelets, beta-blockers, angiotensin-converting enzyme inhibitors (ACEi), calcium channel blockers (CCBs), and statins. The most common comorbidities were ischaemic heart disease (30.2%), diabetes (22.0%), stroke (21.3%), heart failure (18.9%), and cancer (10.9%). By the end of the study period, there was no statistically significant difference between EQ-5D control between baseline and after 3 months in either group. EQ-5D score was noted to be above 0.50 and 0.60 for the control and intervention arms respectively. Mortality rate was not reported for the study. Whilst the study did not single out deprescribing to those medications associated with cardiometabolic conditions, the study did highlight how deprescribing can reduce pill-burden and improve the QoL in the older population residing in care homes and is safe to do so.

### HbA1c targets

Two studies reported on outcomes of glycaemic control following deprescribing interventions. From the two studies, there was no statistical significance noted when comparing the HbA1c levels in those who underwent deprescribing interventions compared with those who did not. Sjoblem et al. found that the HbA1c levels in the deprescribing arm were 5.8% ± 1.1% and in the non-intervention arm were 6.6% ± 1.4% after 6 months of the withdrawal of anti-diabetics., whilst Niznik et al. found that 43.9% (*n* = 3056) of patients were being aggressively overtreated for their diabetes. There was also no significant increase in hypoglycaemic events discussed in those patients within the intervention arm compared to those in the control arm in either study emphasising that there is no statistical significance noted when comparing the HbA1c levels in those who underwent deprescribing interventions compared with those who did not.

### Systolic blood pressure

Four studies assessed the discontinuation of antihypertensive medications in patients > 65 years residing in long-term care facilities. In one study by Gulla et al., SBP was increased following antihypertensive withdrawal from baseline values of 128 ± 19.5 mmHg to 143 ± 25.5 mmHg by month 4; however, by month 9 of follow-up, the SBP had reverted back to baseline values, with a mean of 134 mmHg. In a second study by Song et al., deprescribing was determined to have a statistically significant reduced risk of falls among residents with SBP 80–100 at 17.0% (*n* = 41) in the deprescribing arm and 19.0% (*n* = 375) in the control arm. A further study produced by Vu et al. showed that the study participants had a higher chance of experiencing deprescribing interventions when an SBP < 100 was present and multiple antihypertensives were being used. Further to this, Myers et al. found that the mean blood pressure of the placebo subjects remaining at 12 months (*n* = 26) was (SBP/DBP) 135 ± 4.6 / 74.3 ± 2.9 mmHg which was moderately higher than at baseline 129.3 ± 4.1 / 73.6 ± 2.5 mmHg. A similar change was also seen in the control arm, where the mean blood pressure was 122.1 ± 3.0 / 67.0 ± 1.9 mmHg at baseline increasing to 125.3 ± 3.5 / 69.5 ± 2.2 mmHg at 12 months of follow-up. The cardiothoracic ratio at baseline for the intervention arm was 0.50 ± 0.02, increasing marginally to 0.51 ± 0.02 at 12 months and for the control arm was 0.53 ± 0.01 which remained constant at 12 months. There was no evidence of a significant increased incidence of CHF in those participants withdrawn from diuretic therapy with an incidence of 15.4% (*n* = 6) in the control arm and 5.3% (*n* = 3) in the placebo arm. The results show that although there is a slight increase in SBP noted in one study with an SBP increase of 5.7 mmHg for the exposure group and 2.9 mmHg for the control group, there are no clinically relevant sequelae as a result of deprescribing interventions.

### Mortality

Eight studies assessed the incidence of mortality within their outcomes. Mortality rates found within the antihyperglycaemic studies were deduced to be 8.6% (*n* = 263) for Niznik et al. and 16.0% (*n* = 5) for Sjoblem et al., within the deprescribing arms. Within the antihypertensive deprescribing studies, mortality rates found by Gulla et al. were determined to be 12.0% (*n* = 17) in the intervention arm and 11.0% (*n* = 12) in the control arm, whilst Song et al. found a higher mortality risk in patients with an SBP 101–120 with a mortality rate of 5.4% (*n* = 13) in the deprescribing arm and 2.1% (*n* = 41) in the control arm. For studies discussing the withdrawal of statin therapy, Kutner et al. concluded that the mortality rate was 23.8% (95% CI 3.5 to 10.5%) (*n* = 45) for the intervention arm and 20.3% (95% CI − 3.5 to 10.5%) (*n* = 39) for the control arm, whilst Thorpe et al. found that there was a mortality rate of 23.0% (*n* = 3015) in those who had their medications deprescribed with 57.0% (*n* = 1719) having EoL designation, and 16.0% (*n* = 482) who did not. For the study discussing diuretic withdrawal, the mortality rates for the intervention arm were 20.1% (*n* = 8) and 7.7% (*n* = 3) in the control arm. In a study deprescribing digoxin by Wilkins et al., a mortality rate of 0% was found in their study cohort, whilst in a further study discussing the discontinuation of multiple medications, a mortality rate of 6.3% (*n* = 3) was deduced. These results show us that the mortality rates are slightly higher in those who underwent deprescribing interventions compared with those who did not but were not of such clinical significance so as to raise concern. The mortality rates noted were not always clearly reported, and as such need to be considered with the appropriate clinical judgement. As many of these studies were focusing on those who were on palliative care had an EoL designation or who were living with significant frailty and multimorbidity, it cannot be concretely stated that the slight elevation in mortality rates within the intervention arm compared with the control arm is caused by deprescribing; however, an association may very well be present. Other causes of death should be considered when interpreting the presented results [32].

### Hospitalisations

Two studies assessed the incidence of hospitalisations due to deprescribing interventions. One study by Gulla et al. discussing the description of antihypertensives found that the incidence of hospitalisations was higher in the control group at 13.0% (*n* = 12) compared with the intervention group at 6.0% (*n* = 7), whilst a second study by Song et al., also looking at the description of antihypertensives found that 17.6% (*n* = 42) patients required hospital admission in the deprescribing arm and 13.1% (*n* = 258) in the control arm, and more specifically 17.2 (*n* = 41) were hospitalised due to a fall in the intervention arm and 19.0% (*n* = 375) were admitted in the control arm. These results show us a mixed picture of those requiring hospitalisation following deprescribing interventions, but they do indicate a reduction in falls in the deprescribing arm in those withdrawn from antihypertensives. The results highlight how a reduction in antihypertensive medications in the elderly can have a positive impact on what is one of the biggest causes of hospitalisations in the elderly frail population.

### Complications and adverse events

Four studies assessed adverse events and medication deprescribing complications. One study identified that 6.9% (*n* = 39) of study participants undergoing statin deprescribing experienced cardiovascular events compared with 5.7% (*n* = 11) in the control group. In a study discussing the description of diuretics, it was determined that two patients within the intervention arm developed clinically significant hypertension with two consecutive readings excessing 180/110 mmHg and were re-started on diuretic therapy. In a study by Forman et al., following 60-day follow-up, 1 patient had their digoxin restarted due to a reducing in EF from 60.0 to 50.0%; however, no episodes of congestive heart failure or dysrhythmias were noted. In Wilkins et al. digoxin study, 1 patient developed atrial fibrillation following discontinuation with a ventricular rate of 80–90 beats per minute (bpm) which spontaneously resolved back to normal sinus rhythm for the remaining study period, 1 patient developed dyspnoea on day 10 and as such was initiated on 10 mg furosemide to minimise the symptoms of congestive heart failure, and 1 patient had their digoxin restarted following the development of tachycardia. Whilst some adverse events were noted across the studies, due to limited data and trial participants, it is not clear whether these events were a direct outcome of deprescribing and the results should be interpreted as such.

### Quality of life

Two studies discussed quality of life scores. One study, produced by Kutner et al., found that the Quality of Life (QoL) using the McGill score marginally improved within the deprescribing arm to 7.11 compared with 6.85 in the control arm (total McGill score *p* = 0.04), whilst a further study by Al Aqqad et al. looking at the description of multiple potentially inappropriate medications found that the quality of life within the control arm and the intervention arm, which was assessed using the EuroQol-5 dimension (EQ-5D) assessment tool, found no statistically significant difference between the EQ-5D scores between baseline and follow-up in either group, with an EQ-5D of 0.50 for the control arm and 0.60 for the intervention arm. QoL was seen to increase by a small amount within the studies reporting it, but not by as much as we would expect, this in part could be due to the fact that deprescription of one medication is not enough to improve QoL significantly enough to have an impact on findings, or other signs and symptoms of their ongoing conditions might have impacted the results too.

## Discussion

### Key findings

The results of this systematic review suggest that there is limited high-quality data on deprescribing approaches in the older population with either an end-of-life designation or those residing in long-term care facilities with cardiometabolic conditions and associated multimorbidity and this is the first systematic review determining the adverse events and outcomes associated with deprescribing in this cohort of patients. Deprescribing approaches that have been studied within these 13 papers have included the complete withdrawal of medications and the reduction in dose of a medication, with the majority of studies focussing on the complete withdrawal of medications. The success of the deprescribing interventions varied from 27.0 to 94.7%.

For papers discussing the deprescription of antihyperglycaemics, antihypertensives, and diuretics, there were no statistical significances noted between HbA1c levels and systolic blood pressure readings in the control and intervention arms, respectively, with no significant difference in the mortality rates found between the two groups. The quality of life of individuals who had their statins deprescribed did not change significantly when compared with those individuals who were continued on usual care, and no comment was made regarding significant cardiovascular events in those patients who had their aspirin, or their digoxin discontinued. Mortality rates were noted to be similar across both arms in many studies where mortality was noted as a relevant outcome; however, it was not stated in any of the studies whether these mortality rates were directly linked with deprescribing efforts, and as such the results should be interpreted with care. When considering this, it is evident that further research into mortality outcomes associated with deprescribing are needed to better evaluate the potential risks of deprescribing cardiometabolic conditions against the proposed benefits, due to the limited evidence available for synthesis within this review. Nevertheless, given that medications are usually prescribed to prevent harm including death, the absence of worse mortality outcomes in the deprescribing population is reassuring to both patients and healthcare professionals. QoL indicators were not routinely reported, however in the studies that did report it, the scores were only marginally improved in the deprescribing arm compared with the intervention arm. Considering the populations that were being sampled, other possible reasons why QoL indicators were not improved as much as was initially predicted could include that QoL was already relatively low given current clinical frailty and multimorbidity status, or that deprescribing one medication when latest reports have highlighted that the average individual aged > 65 years old is taking > 7 medications [30], might not have enough of an impact on reducing pill burden and associated tests to improve the QoL of older people.

It is also worth noting that there may be bias between the groups as not all studies reporting mortality were RCTs. It was evident from the studies looking at multiple medications that decreasing the effects of polypharmacy is required and can be done when each patient’s medications are carefully revised by an appropriate healthcare clinician and can be done in those with significant cardiometabolic and diabetic comorbidities. From the studies, we can deduce that patients are more likely to have deprescribing interventions carried out when they have a documented end-of-life status and that there is a desperate need to undertake more rigorous clinical trials into the deprescribing process.

With respect to the possibilities of confounders, i.e., whether an observed exposure or predictor is causally associated with one of the above outcomes, we are unable to concretely state whether any exist for the patients included within the study. As the majority of studies were observational studies, the possibility of selection bias is high in that patients who are deprescribed may differ from those who do not, meaning there is a high possibility of confounders between deprescribing and adverse events and outcomes including mortality and hospitalisations. The only way to assess these relationships accurately would be to carry out RCTs where patients are randomised to deprescribing to reduce selection bias. Three RCTs were noted but were found to be of limited evidence. As such, the review should be assessed with a degree of scepticism about its truth. It is also worth noting, that due to the limited number of studies it is not clear which deprescribing approach is the most effective within this cohort of patients, whilst many studies described the complete withdrawal of a medication, there is no concrete evidence to suggest that this is the best approach to improve the quality of life of older patients in their final years of life.

### Comparison with previous studies

To the best of the authors’ knowledge, no other study has been produced comparing the deprescription of multiple medications for the therapeutic interventions of cardiometabolic conditions in the older population in those residing in long-term care facilities or with an end-of-life designation. Through the data base searches, six reviews were highlighted that bore similarities to this review including studies that looked at the identification and resolution of medications in long-term care facilities, the deprescription of central nervous system medication, and the withdrawal of antihypertensives and cardiovascular medications in older patients who were not frail with significant cardiometabolic multimorbidities.

Previous studies have described the deprescription of individual medications under one condition topic; however, this review has a much broader approach and classifies the conditions under the umbrella term of cardiometabolic conditions.

### Strengths and limitations

No other systematic review identified by the authors compares the deprescribing of multiple medications surrounding those patients with cardiometabolic conditions and multimorbidity residing in long-term care facilities or with an end-of-life designation. The literature search was comprehensive and considered multiple databases, yielding a total of 13 papers relevant to the research topic. A good range of studies was also included in the review comprising observational studies and RCTs. The data found was limited in quantity and heterogeneous, hence data was unable to be collated and analysed in the manner originally planned, however the evidence within the studies was able to be grouped together and narratively analysed according to the themes set out to be identified, in line with the protocol submitted (CRD42021291061). The duration of follow-up varied, but at most covered 12 months, meaning the produced evidence has limited impact on long-term follow-up conclusions. The adverse events and outcomes highlight the importance of regular follow-up consultations with the patient and re-evaluation of the patient’s clinical status as this could heavily influence the patient’s management plan. The produced data does show that deprescribing interventions are feasible amongst this cohort of patients which shows promise for future research and clinical trials.

The limitations of this study focus on the lack of comparable data contained within the studies and the heterogeneous nature of the data meaning that a meta-analysis was not able to be undertaken. Whilst a range of study designs were included in the review, they were not always of a high quality and mostly comprised of observational studies, which can result in selection bias [33]. A large number of the studies did not have a good duration of follow-up, mostly comprising of short follow-up periods of 60 days to 3 months which does not enable all possible outcomes to be assessed, and as such the results should be interpreted with this in mind. Across the studies, mortality was not routinely noted and reported upon, which is something that reduces the overall impact of the studies and their overall results. Further studies looking into deprescribing in the older, frail, multimorbid patient should consider monitoring for mortality rates across both arms of the study to enhance the power of their results. A further limitation to note is that only papers written in the English language were included within the systematic review due to translational services being unavailable at the time the review was conducted. The data that was provided within the studies did enable some comparisons to be made with regard to deprescribing success rates and adverse outcomes and complications which could be grouped together and narratively synthesised.

### Implication of findings

The main focus of management of older patients with cardiometabolic conditions living with frailty, multimorbidity, or an EoL designation is the reduction of acute adverse outcomes, and to reduce the use of medications that reduce long-term outcomes such as lowering cholesterol levels or keeping patients HbA1c within a tight glycaemic range (to less than 7.0%). It is becoming clear from the evidence being produced in current studies that this over treatment and tight control is no longer needed, and the shift of management should be towards improving the quality of life of patients and managing acute problems such as pain management. Studies have shown that the number of adverse events such as cardiovascular events associated with statin deprescribing is only marginally larger within the deprescribing cohort compared with the control cohort [7, 8], and SBP only marginally raises in those undergoing deprescribing efforts associated with antihypertensive withdrawal versus those who continue on long-term therapy, but were found to stabilise after 12 months of no treatment. When considering those with multimorbidity, it is important to reflect upon their past, observe the present, and predict the future, to ensure patient-centred goals are at the forefront of management to improve QoL whilst ensuring mortality is being reduced [4]. This systematic review has highlighted that the deprescription of cardiometabolic medications can be safely carried out in this sample population and has the potential to improve markers such as QoL and patient-centred goals. However, the review has highlighted that more research is needed to look into QoL indicators, mortality and morbidity outcomes, and adverse events and outcomes.

Other systematic reviews focusing on deprescribing, not focusing on cardiometabolic conditions, can be used in conjunction with this review to provide a more thorough overview of deprescribing as a whole. A review of randomised control trials looking into the reduction of polypharmacy identified that mortality was reduced when using deprescribing interventions [31], whilst other reviews have highlighted that deprescribing in the elderly population can improve cognitive function and can reduce falls [30]. Overall, studies have demonstrated that no harm has come to patients included in deprescribing trials.

## Conclusion

Improving the quality of life of older patients should be the mainstay of care when considering older patients with end-of-life designations or those who are residing in long-term care facilities. With predisposing factors such as frailty, advancing age, polypharmacy, prescribing cascades, and multi-morbidity, these older patients should be managed from a more holistic approach, focusing on their quality of life, as intensive management in these patients is associated with a higher risk of adverse events such as hypoglycaemia and falls [34]. This systematic review highlights the importance of deprescribing in this population and shows that it is feasible with negligible significant effects on the adverse outcomes such as hospitalisation episodes, falls, and cardiovascular events. Whilst guideline bodies have identified the need for individualised targets for patients, such as individualised HbA1c targets, or cholesterol levels, current guidelines suggest that the approach should be patient-focused considering the preference of the patient, comorbidities, projected life expectancy, and the severity of their condition-related complications [35, 36]. However, given the heterogeneity of patients with cardiometabolic conditions, further research and RCTs are needed to provide safe individualised targets for these patients and identify the most appropriate deprescribing approaches for this cohort of patients. In turn, this would allow for a meta-analysis to be conducted offering more in-depth knowledge in this field and a broader review of the possible evidence to support guidance in clinical practice.

Even though limited and low-quality data suggests that the quality of life did not significantly improve with de-prescribing interventions, the absence of significant worsening of surrogate and clinical outcomes in the population of people in long-term care facilities towards the end of life is a reassuring finding. Further studies on this topic focusing on quality of life and mortality outcomes are therefore needed. It can be concluded from the included studies that no significant adverse events were noted as a result of deprescribing interventions when considering older patients with cardiometabolic conditions with end-of-life designations, frailty, or multimorbidity residing in long-term care facilities and that the process itself is feasible for this cohort of patients.

### Supplementary Information

Below is the link to the electronic supplementary material.

Supplementary file1 (DOCX 56 KB)

## Figures and Tables

**Fig. 1:**
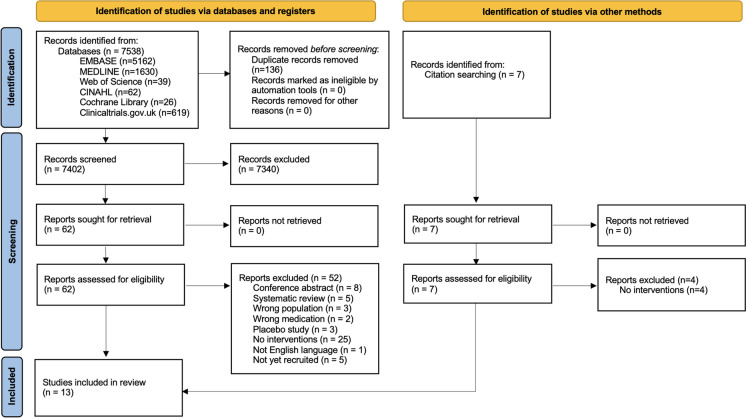
PRISMA flow chart

## Data Availability

The authors confirm that the data supporting the findings of this study are available within the article and/or its supplementary materials.

## Appendix

Figure 1

Tables 1 and 2

**Table 1:** Baseline characteristics

Lead author	Location	Study design	Baseline population	Mean age	% Female	Duration of follow-up	Total participants
Al Aqqad, 2014	Malaysia	Prospective cohort	Nursing home residents taking > 1 medication	77.7	60.7	3 months	211
Forman, 1991	USA	Pre-post study	Nursing home residents with a normal ejection fraction or in normal sinus rhythm who are > 65 years old	87	70	60 days	14
Gulla, 2018	Norway	Cluster-randomised control trial	Nursing home residents with hypertension	87.2	70	9 months	295
Kutner, 2015	USA	Multi-centre, controlled, unblinded, pragmatic randomised clinical trial	Elderly patients with a life-limiting condition using statins	74.1	44.9	12 months	381
Myers, 1982	Canada	Randomised clinical trial	Nursing home residents with congestive heart failure using diuretics	82.2	22.1	12 months	77
Niznik, 2020	USA	Retrospective cohort	Veterans with T2DM	77.6	0.9	90 days	3056
Onder, 2019	Israel and Europe	Longitudinal multi-centre cohort	Elderly patients on > 5 medications	84	74.9	12 months	1843
Sjoblem, 2008	Sweden	Prospective cohort with controls	Elderly patients with T2DM and a HbA1c < 6.0%	84.1	59.3	6 months	98
Song, 2018	USA	Retrospective cohort	Elderly patients residing in long-term care with falls	80.5	2.3	30 days	2212
Springer, 2020	USA	Retrospective cohort	Elderly patients with a history of cardiovascular disease requiring aspirin in nursing home facilities	75	1.1	90 days	13,844
Thorpe, 2020	USA	Retrospective cohort	Elderly Veterans with coronary artery disease, stroke, or diabetes, using statins	79	1.1	91 days	13,110
Vu, 2021	USA	Retrospective cohort	Elderly Veterans with hypertension in nursing homes	79	1.3	30 days	10,574
Wilkins, 1985	USA	Prospective cohort	Nursing home residents requiring digitalis	84.9	84.2	4 months	19

**Table 2:** Table of reported outcomes

Lead author	Selection of patients	Deprescribing approach	Control	Intervention to control ratio	Deprescribing rate	Therapeutic control	Outcomes and complications	Mortality
Al Aqqad, 2014	Nursing home residents aged > 65 taking at least 1 medication identified using the STOPP criteria	PIMS identified using STOPP criteria and reassessed after 3 months	No control	211:0	5.1% reduction in PIMs	No statistically significant difference between EQ-5D control between baseline and after 3 months in either group	EQ-5D score above 0.50 and 0.60 for the control and intervention respectively	NR
Forman, 1991	Patients aged > 65 in two urban nursing homes taking digoxin	Digoxin stopped when found to be in NSR or to have a normal echo LV EF	No control	14:0	92.8% success rate in withdrawal of digoxin	28.6% (*n* = 4) had an increased EF No patients had an LV EF drop below 50%	7.14% (*n* = 1) had an LV EF drop from 60 to 50% None showed signs of clinical deterioration—no episodes of CHF or dysrhythmias	NR
Gulla, 2018	Elderly nursing home residents aged > 65 years old taking antihypertensive treatment	Patients > 65 years old with antihypertensive treatment were randomly allocated to either systematic medication review or continued therapy with 9-month follow-up	Received usual care	164:131	51.2% success rate in withdrawal of antihypertensives	SBP increased by month 4 and then reduced to baseline levels by month 9 in the intervention arm	By month 4: 5% (*n* = 7) in the intervention arm and 13% (*n* = 14) in the control were admitted to hospital By month 9: 6% (*n* = 7) in the intervention arm and 13% (*n* = 12) in the control were admitted to hospital	By month 4: 12% (*n* = 19) in the intervention arm and 13% (*n* = 17) in the control arm By month 9: 12% (*n* = 17) in the intervention arm and 11% (*n* = 12) in the control arm
Kutner, 2015	Adults with an estimated life expectancy of between 1 month and 1 year, statin therapy for 3 months or more with no recent active cardiovascular disease	Participants were randomised using block randomisation to either discontinue or continue statin therapy and monitored every month for 1 year	Received usual care	189:192	NR	QoL McGill Score 7.11 for the intervention arm and 6.85 for the control arm 9-item Edmonton Symptom Assessment Scores lower in intervention arm, 7.11, compared with 6.85 in control arm	QoL McGill Score 7.11 for the intervention arm and 6.85 for the control arm 13 cardiovascular events in the intervention arm and 11 in the control arm	23.8% (*n* = 45) in the intervention arm 20.3% (*n* = 39) in the control arm
Myers, 1982	Elderly patients in two geriatric institutions with current diuretic use not concomitantly using digoxin, for congestive heart failure or hypertension	Randomly allocated to either continue diuretic therapy or given a placebo and followed at regular intervals	Placebo	38:39	74.4% success rate after 12 months	No difference between baseline SBP and DBP for both the intervention and control groups including supine, sitting and erect with a decreased change in SBP and DBP for postural hypotension in the intervention group. No significant difference in heart rates across both groups	15.38% (*n* = 6) in the control arm and 5.26% (*n* = 2) in the placebo arm developed CHF 5.65% (*n* = 2) in the placebo group developed hypertension	7.69% (*n* = 3) in the in the control group 20.05% (*n* = 8) in the intervention group (*n* = 1 with CHF, *n* = 7 from associated causes)
Niznik, 2020	Elderly Veterans with diabetes and LLE/AD with HbA1c measured within 90 days of admission using antihyperglycaemics with HbA1c < 7.5% and receiving > 1 diabetes medication	Switching from using both short-acting and long-acting insulin to just long-acting insulin or discontinuing insulin altogether	No control	3056:0	45.5% success rate after 90 days Short acting insulin 20.7%, sulfonylureas 9.7%, basal insulin 7.2% and non-insulin/non-sulfonylurea agents 5.6%	Respective cumulative incidence of deintensification at 15, 30, 60, and 90 days was 22.0%, 30.1%, 38.0%, and 45.5%, respectively	Increased likelihood of discontinuation with documented end of life status	8.6% (*n* = 263)
Onder, 2019	Elderly patients residing in nursing homes taking > 5 medications	A reduction in the number of medications over the study period across 57 nursing homes	Received usual care	658:1185	35.7% (*n* = 658) residents	Higher rates of deprescribing in those with cognitive impairment, increased numbers of medications used at baseline and a residing geriatrician at the facility. Lower rates observed with female gender, heart failure and cancer	Reduction in use of antiplatelets, beta-blockers, ACEi, CCBs, and statins across 6- and 12-month follow-up. Increased use of laxatives and analgesics used in control arm	NR
Sjoblem, 2008	Elderly patients with a HbA1c < 6.0% using antihyperglycaemics, as individual or combination treatment, participated in medication discontinuation	Blood plasma glucose levels were taken on 3 consecutive days prior to drug withdrawal	No alteration to diabetes medication	32:66	75.0% (*n* = 24) success rate 3 months after drug withdrawal	HbA1c levels in the deprescribing arm were 5.8% ± 1.1% and 6.6% ± 1.4% in the non-intervention arm after 6 months		16% (*n* = 5) in the deprescribing arm and 21% (*n* = 14) in the control arm
Song, 2018	Nursing home long stay residents (> 91 days) who are > 65 years old with evidence of overaggressive antihypertensive treatment and an index fall followed up for 30 days	Deintensification of antihypertensive treatment using propensity score methods (PSM)	Received usual care	329:1973	NR	17.2% (*n* = 41) falls in the intervention arm and 19.0% (*n* = 375) in the control arm 17.6% (*n* = 42) in the intervention arm and 13.1% (*n* = 258) in the control arm was admitted to hospital	Lower risk of recurrent falls among residents with SBP 80–100 and higher risk of death in patients with SBP 101–120 in the intervention arm	5.4% (*n* = 13) in the intervention arm and 2.1% (*n* = 41) in the control arm
Springer, 2020	Older patients > 65 years old with LLE/AD admitted for > 7 days who had a history of CAD, stroke, or TIA and used aspirin within week 1 of admission	14 days of aspirin discontinuation in those using for secondary cardiovascular prevention	No control	13,844:0	27.0% for the full sample—34% in those with an LP and 24% in those without an LP	27% reduction in aspirin use	Diabetes and congestive heart failure were associated with a lower rate of discontinuation	NR
Thorpe, 2020	Veterans > 65 years old with CAD, stroke, or diabetes who met criteria for their LLE/AD on admission to a VA CLC centre and received statins within week 1 following admission	Statins discontinued	No control	13,110:0	30.9% (*n* = 4064) discontinuation rate in first 90 days following CLC admission	Cumulative incidence of discontinuation higher in residents with an EOL designation/hospice care at admission—52% (*n* = 2113) with EOL designation versus 25% (*n* = 1016) without EOL designation	Prompts for deprescribing include EOL designation, hospice use and individual clinical factors	23% (*n* = 3015) mortality, 57% (*n* = 1719) had EOL designation or hospice care and 16% (*n* = 482) who did not
Vu, 2021	Older veterans with LLE/AD and HTN admitted to VA nursing homes receiving at least 1 antihypertensive class and mean daily SBP < 120	Antihypertensives deprescribed (dose reduced or discontinued) for > 7 days	No control	10,574:0	40.9% (*n* = 4335) discontinuation at 30 days		Multiple antihypertensive use and SBP < 100 mmHg was associated with a higher risk of deprescribing	NR
Wilkins, 1985	Nursing home patients using digoxin in NSR with no evidence of CHF	Discontinuation of digoxin followed up for 4 months	No control	19:0	94.7% (*n* = 18) success rate of deprescribing digoxin	81.25% (*n* = 16) showed no changes in clinical status. 5.26% (*n* = 1) patient developed AF with VR = 80–90 bpm on day 5 and then converted back to NSR spontaneously for the remainder of the study 5.26% (*n* = 1) patient developed dyspnoea on day 10	Digoxin restarted in 1 patient who developed sinus tachycardia Furosemide 20 mg was started in one patient who developed clinical features of CHF	0% (*n* = 0) mortality rate
