# Supplementary material for: Deprescribing in cardiometabolic conditions in older patients: a systematic review
Source: GeroScience. 2023 Jul 5;45(6):3491–512. doi: 10.1007/s11357-023-00852-z (PMC10643631; doi:10.1007/s11357-023-00852-z)
Supplement: Supplementary file 1 — Supplementary file1 (DOCX 56 KB) [file 11357_2023_852_MOESM1_ESM.docx]

**Deprescribing in Cardiometabolic Conditions in Older Patients: A Systematic Review**

**GeroScience**

Elizabeth Hickman^1^, Mansha Seawoodharry^1^ Clare Gillies^1^, Kamlesh Khunti^1^, Samuel Seidu^1^

^1^Diabetes Research Centre, Leicester Diabetes Centre, University of Leicester, Leicester General Hospital, Gwendolen Road, Leicester, LE5 4WP, UK

Correspondence to: Elizabeth Hickman

Diabetes Research Centre, University of Leicester,

Leicester General Hospital, Gwendolen Road,

Leicester, LE5 4WP, UK

Email: [emh35@leicester.ac.uk](mailto:emh35@leicester.ac.uk)

## **Supplementary Material**

Table S1: PRISMA checklist

Table S2: MOOSE Checklist

Figure S1: Search strategies

Table S3: Eligibility Criteria

Table S4: Study quality assessment using the Newcastle-Ottawa tool for Cohort Studies

Table S5: Study quality assessment using the Newcastle-Ottawa tool for RCTs

Table S6: NIH Bias tool for Pre-Post Studies

## **Table S1: PRISMA Guidelines**

| **Section and Topic** | **Item #** | **Checklist item** | **Location where item is reported** |
| --- | --- | --- | --- |
| **TITLE** | | |  |
| Title | 1 | Identify the report as a systematic review. | 1 |
| **ABSTRACT** | | |  |
| Abstract | 2 | See the PRISMA 2020 for Abstracts checklist. | 2 |
| **INTRODUCTION** | | |  |
| Rationale | 3 | Describe the rationale for the review in the context of existing knowledge. | 3 |
| Objectives | 4 | Provide an explicit statement of the objective(s) or question(s) the review addresses. | 3 |
| **METHODS** | | |  |
| Eligibility criteria | 5 | Specify the inclusion and exclusion criteria for the review and how studies were grouped for the syntheses. | 4 |
| Information sources | 6 | Specify all databases, registers, websites, organisations, reference lists and other sources searched or consulted to identify studies. Specify the date when each source was last searched or consulted. | 4 |
| Search strategy | 7 | Present the full search strategies for all databases, registers and websites, including any filters and limits used. | Supplementary S4 |
| Selection process | 8 | Specify the methods used to decide whether a study met the inclusion criteria of the review, including how many reviewers screened each record and each report retrieved, whether they worked independently, and if applicable, details of automation tools used in the process. | 4, 5 |
| Data collection process | 9 | Specify the methods used to collect data from reports, including how many reviewers collected data from each report, whether they worked independently, any processes for obtaining or confirming data from study investigators, and if applicable, details of automation tools used in the process. | 4, 5 |
| Data items | 10a | List and define all outcomes for which data were sought. Specify whether all results that were compatible with each outcome domain in each study were sought (e.g. for all measures, time points, analyses), and if not, the methods used to decide which results to collect. | 6, supplementary tables S1 and S2 |
|  | 10b | List and define all other variables for which data were sought (e.g. participant and intervention characteristics, funding sources). Describe any assumptions made about any missing or unclear information. | 6 |
| Study risk of bias assessment | 11 | Specify the methods used to assess risk of bias in the included studies, including details of the tool(s) used, how many reviewers assessed each study and whether they worked independently, and if applicable, details of automation tools used in the process. | 6, supplementary tables S4, S5, and S6 |
| Effect measures | 12 | Specify for each outcome the effect measure(s) (e.g. risk ratio, mean difference) used in the synthesis or presentation of results. | N/A |
| Synthesis methods | 13a | Describe the processes used to decide which studies were eligible for each synthesis (e.g. tabulating the study intervention characteristics and comparing against the planned groups for each synthesis (item #5)) | Supplementary tables S1 and S2 |
|  | 13b | Describe any methods required to prepare the data for presentation or synthesis, such as handling of missing summary statistics, or data conversions. | N/A |
|  | 13c | Describe any methods used to tabulate or visually display results of individual studies and syntheses. | Supplementary material |
|  | 13d | Describe any methods used to synthesize results and provide a rationale for the choice(s). If meta-analysis was performed, describe the model(s), method(s) to identify the presence and extent of statistical heterogeneity, and software package(s) used. | 5, 6, 7, 8 |
|  | 13e | Describe any methods used to explore possible causes of heterogeneity among study results (e.g. subgroup analysis, meta-regression). | N/A |
|  | 13f | Describe any sensitivity analyses conducted to assess robustness of the synthesized results. | N/A |
| Reporting bias assessment | 14 | Describe any methods used to assess risk of bias due to missing results in a synthesis (arising from reporting biases). | 6 |
| Certainty assessment | 15 | Describe any methods used to assess certainty (or confidence) in the body of evidence for an outcome. | N/A |
| **RESULTS** | | |  |
| Study selection | 16a | Describe the results of the search and selection process, from the number of records identified in the search to the number of studies included in the review, ideally using a flow diagram. | 6, supplementary S2 |
|  | 16b | Cite studies that might appear to meet the inclusion criteria, but which were excluded, and explain why they were excluded. | Supplementary S2 |
| Study characteristics | 17 | Cite each included study and present its characteristics. | 6, 7, supplementary table S2 |
| Risk of bias in studies | 18 | Present assessments of risk of bias for each included study. | Supplementary tables S4, S5, and S6 |
| Results of individual studies | 19 | For all outcomes, present, for each study: (a) summary statistics for each group (where appropriate) and (b) an effect estimate and its precision (e.g. confidence/credible interval), ideally using structured tables or plots. | 7, 8, 9, 10 |
| Results of syntheses | 20a | For each synthesis, briefly summarise the characteristics and risk of bias among contributing studies. | 7, 8, 9, 10, 11 |
|  | 20b | Present results of all statistical syntheses conducted. If meta-analysis was done, present for each the summary estimate and its precision (e.g. confidence/credible interval) and measures of statistical heterogeneity. If comparing groups, describe the direction of the effect. | 7, 8, 9, 10 |
|  | 20c | Present results of all investigations of possible causes of heterogeneity among study results. | N/A |
|  | 20d | Present results of all sensitivity analyses conducted to assess the robustness of the synthesized results. | N/A |
| Reporting biases | 21 | Present assessments of risk of bias due to missing results (arising from reporting biases) for each synthesis assessed. | 11, 12 |
| Certainty of evidence | 22 | Present assessments of certainty (or confidence) in the body of evidence for each outcome assessed. | 11, 12 |
| **DISCUSSION** | | |  |
| Discussion | 23a | Provide a general interpretation of the results in the context of other evidence. | 11 |
|  | 23b | Discuss any limitations of the evidence included in the review. | 11, 12 |
|  | 23c | Discuss any limitations of the review processes used. | 11, 12 |
|  | 23d | Discuss implications of the results for practice, policy, and future research. | 12, 13 |
| **OTHER INFORMATION** | | |  |
| Registration and protocol | 24a | Provide registration information for the review, including register name and registration number, or state that the review was not registered. | 2 |
|  | 24b | Indicate where the review protocol can be accessed, or state that a protocol was not prepared. | 2, 3, 4 |
|  | 24c | Describe and explain any amendments to information provided at registration or in the protocol. | N/A |
| Support | 25 | Describe sources of financial or non-financial support for the review, and the role of the funders or sponsors in the review. | 13 |
| Competing interests | 26 | Declare any competing interests of review authors. | 13 |
| Availability of data, code and other materials | 27 | Report which of the following are publicly available and where they can be found: template data collection forms; data extracted from included studies; data used for all analyses; analytic code; any other materials used in the review. | N/A |

*From:*  Page MJ, McKenzie JE, Bossuyt PM, Boutron I, Hoffmann TC, Mulrow CD, et al. The PRISMA 2020 statement: an updated guideline for reporting systematic reviews. BMJ 2021;372:n71. doi: 10.1136/bmj.n71

**Table S2: MOOSE Checklist**

| **Criteria** | | **Brief description of how the criteria were handled in the meta-analysis** |
| --- | --- | --- |
| **Reporting of background should include** | |  |
| √ | Problem definition | Identifying outcomes of deprescribing interventions in elderly patients with diabetes with and without cardiometabolic comorbidities residing in long term care facilities. Information regarding the benefits and harms of deprescribing in this elderly population is unclear. Specific outcomes include complications, mortality, hospitalisation, falls, increases in laboratory markers, patient satisfaction and quality of life. A systematic review of published literature has been conducted with the outlined specific outcomes evaluated with respect to the deintensification of anti-hyperglycaemic medications and other therapies in the elderly population (those >65 years old) with type two diabetes with or without cardiometabolic conditions. |
| √ | Hypothesis statement | Associated adverse events and outcomes with deprescribing interventions in the older populations with end-of-life designation or in long term facilities |
| √ | Description of study outcomes | Hospitalisations  Complications (e.g., hyperglycaemia, diabetic ketoacidosis (DKA), hyperglycaemic hyperosmolar state (HHS), hypotension, hypertension, hyperlipidaemia, etc.)  Mortality  Falls  Patient satisfaction  Quality of life  Deprescribing success rates |
| √ | Type of exposure or intervention used | Deprescribing interventions to include, complete withdrawal, reduction in dose, tapering of dose or switching to an alternative medication, of at least 1 medication |
| √ | Type of study designs used | Observational studies including cross-sectional, prospective cohort, retrospective cohort, prospective case control, retrospective case control, pre-post studies and case-cohort  Clinical trials including randomised control trials (RCTs), non-randomised control trials, cluster, and pragmatic control trials |
| √ | Study population | Elderly patients aged >65 years old  Type 2 diabetes with or without cardiometabolic conditions  Cardiometabolic conditions (e.g., stroke, ischaemic heart disease, hypertension etc.)  On therapeutic dose medications for either diabetes or cardiometabolic condition (e.g., anti-hyperglycaemics, anti-hypertensives, statins, aspirin etc.)  Deprescribing approaches reported  Reported outcomes including, mortality, complications, hospital admissions, quality of life and patient satisfactions  Over 65 years old or if the mean age of study participants is greater than 65 years old or if data for the over 65 population can be extracted from the paper |
| **Reporting of search strategy should include** | |  |
| √ | Qualifications of searchers | Elizabeth Hickman, MSc. Mansha Seawoodharry, PhD |
| √ | Search strategy, including time period included in the synthesis and keywords | Time period: from inception of MEDLINE and EMBASE to March of 2022.  Search strategy: detailed search strategy can be found in Appendix 1.2 |
| √ | Databases and registries searched | MEDLINE, EMBASE, clinicaltrials.gov.uk, Cochrane databases, Web of Science, CINAHL |
| √ | Search software used, name and version, including special features | OvidSP was used to search MEDLINE and EMBASE  EndNote 9 was used to manage references |
| √ | Use of hand searching | Reference lists and bibliographies were manually/hand searched for additional studies |
| √ | List of citations located and those excluded, including justifications | The literature search process is outlined in the PRISMA flow chart seen in Appendix 1.4. The citation list for excluding studies is available upon request. |
| √ | Method of addressing articles published in languages other than English | Language was restricted to English only due to having no translator services available. |
| √ | Method of handling abstracts and unpublished studies | We did not include unpublished or abstract only publications |
| √ | Description of any contact with authors | We did not need to contact any authors for this review |
| **Reporting of methods should include** | |  |
| √ | Description of relevance or appropriateness of studies assembled for assessing the hypothesis to be tested | The inclusion and exclusion criteria has been outlined in Appendix 1.3. They have also been described in the Methods section 2.3.2 |
| √ | Rationale for the selection and coding of data | The data that was extracted for inclusion was in accordance with the outlined study population and characteristics, study design, study type, exposure of interest and outcomes |
| √ | Assessment of confounding | N/A |
| √ | Assessment of study quality, including blinding of quality assessors; stratification or regression on possible predictors of study results | Assessment of the study quality was carried out using the Newcastle Ottowa Scale (9-star scale) for cohort studies and the Newcastle Ottowa Scale (9-star scale) for randomised control trials, and the NIH quality assessment tool (12-question scale) for pre-post studies with no control group. The NOS scale looks at 3 main categories, namely selection (representiveness of population), comparability (accounts for confoundment) and outcome. |
| √ | Assessment of heterogeneity | Not enough data to assess heterogeneity of studies |
| √ | Description of statistical methods in sufficient detail to be replicated | N/A |
| √ | Provision of appropriate tables and graphics | S3 (table 1) showing baseline characteristics, S4 (table 2) showing , S5 (table 3) showing NOS for RCTs, S6 (table 4) showing NOS for , S7 (table 5) showing NIH bias tool for pre-post studies |
| **Reporting of results should include** | |  |
| √ | Graph summarizing individual study estimates and overall estimate | N/A |
| √ | Table giving descriptive information for each study included | S3 and S4 (tables 1 and 2 respectively) of supplementary material |
| √ | Results of sensitivity testing | N/A |
| √ | Indication of statistical uncertainty of findings | 95% confidence intervals have been used where appropriate |
| **Reporting of discussion should include** | |  |
| √ | Quantitative assessment of bias | The review includes published data and as such there is limited scope to produce a quantitative assessment of bias |
| √ | Justification for exclusion | Studies were assessed using the pre-defined inclusion and exclusion criteria (seen in Appendix 1.3) and outlined in the methods section of this report (section 2.3.2) |
| √ | Assessment of quality of included studies | Discussion around the assessment of the quality of included studies has been briefly approached in the study quality (section 2.4.3) (Appendix 1.9, 1.10 and 1.11) of this report |
| **Reporting of conclusions should include** | |  |
| √ | Consideration of alternative explanations for observed results | There are limited studies included in this review due to a limited number of publications in this research area, therefore the results should be reviewed with caution. The studies included are also limited by study quality and again the results should be reviewed and interpreted with caution |
| √ | Generalization of the conclusions | Discussions for conclusions can be seen in the key findings section. Deprescribing interventions are feasible in the older population with cardiometabolic conditions who have an end-of-life designation or are residing in long term care facilities with multimorbidity. |
| √ | Guidelines for future research | Definitive clinical trials (RCTs) are required in this research area to consolidate the findings found in this review along with other published reviews in this field. RCTs are required to assess the impacts of deprescribing on outcomes such as mortality, to mitigate any selection bias seen in the observational studies included within this review. |
| √ | Disclosure of funding source | No additional funding was provided for this systematic review. The PhD is funded by NIHR and the University of Leicester, for which this systematic review was undertaken |

**Figure S1: Search Strategies**

**Figure S1: 1.1 Ovid MEDLINE(R) ALL <1946 to March 14, 2022>**

1 deprescription.mp. or exp Deprescriptions/ 882

2 deprescrib*.mp. 1163

3 deprescrip*.mp. 915

4 exp Drug Tapering/ 263

5 (reduced or reduces or reducing or reduction).mp. 3282541

6 (ceased or ceasing or cessation*).mp. 109020

7 discontinu*.mp. 140871

8 stop*.mp. 148438

9 withdraw*.mp. 144773

10 taper*.mp. 24193

11 deintensification*.mp. 174

12 (Deprescri* or "de-prescri" or "unprescri*" or "cease" or "ceasing" or "withdraw" or "discontinu" or "stop" or "intermittent").mp. 175859

13 1 or 2 or 3 or 4 or 5 or 6 or 7 or 8 or 9 or 10 or 11 or 12 3796734

14 exp Diabetes Mellitus/ or exp Diabetes Mellitus, Type 2/ 471537

15 "non-insulin dependent diabetes".mp. 8700

16 NIDDM.mp. 6974

17 T2DM.mp. 26929

18 DM2.mp. 2375

19 "type 2 diabete*".mp. 149047

20 "type ii diabete*".mp. 9292

21 "type two diabete*".mp. 221

22 (type 2 adj3 diabet*).mp. [mp=title, abstract, original title, name of substance word, subject heading word, floating sub-heading word, keyword heading word, organism supplementary concept word, protocol supplementary concept word, rare disease supplementary concept word, unique identifier, synonyms] 211809

23 (type two adj3 diabet*).mp. [mp=title, abstract, original title, name of substance word, subject heading word, floating sub-heading word, keyword heading word, organism supplementary concept word, protocol supplementary concept word, rare disease supplementary concept word, unique identifier, synonyms] 264

24 (type ii adj3 diabet*).mp. [mp=title, abstract, original title, name of substance word, subject heading word, floating sub-heading word, keyword heading word, organism supplementary concept word, protocol supplementary concept word, rare disease supplementary concept word, unique identifier, synonyms] 11737

25 exp Cardiovascular Diseases/dt, ep, tu [Drug Therapy, Epidemiology, Therapeutic Use] 545282

26 cardiometabolic.mp. 15821

27 exp Hypertension/ or hypertension.mp. 530477

28 exp Hemorrhagic Stroke/ or exp Stroke/ or exp Embolic Stroke/ or stroke.mp. or exp Thrombotic Stroke/ or exp Ischemic Stroke/ 358782

29 "cerebrovascular accident*".mp. 7777

30 exp Ischemic Attack, Transient/ or "transient isch*emic attack*".mp. 30518

31 "heart failure".mp. or exp Heart Failure/ 233800

32 exp Coronary Artery Disease/ or exp Myocardial Infarction/ or "coronary artery disease*".mp. or exp Myocardial Ischemia/ or exp Coronary Disease/ 488460

33 exp Peripheral Arterial Disease/ or exp Peripheral Vascular Diseases/ or "peripheral artery disease*".mp. 59754

34 exp Arterial Occlusive Diseases/ or "arterial disease*".mp. 264853

35 ("arteriosclerosis" or "atherosclerosis").mp. 185775

36 exp Atherosclerosis/ or exp Arteriosclerosis/ or exp Plaque, Atherosclerotic/ or Atherosclerotic.mp. 228653

37 "heart attack".mp. 4863

38 ("NSTEMI" or "STEMI" or "ACS").mp. 39108

39 "acute coronary syndrome".mp. or exp Acute Coronary Syndrome/ 32765

40 exp Angina, Unstable/ or exp Angina Pectoris/ or angina.mp. 72727

41 (angina adj3 (stable or unstable)).mp. 25135

42 exp Dyslipidemias/ or dyslipid*emia.mp. or exp Hyperlipidemias/ 109727

43 exp Hypercholesterolemia/ or hypercholesterol*emia.mp. 47502

44 "lipid metabolism disorder*".mp. or exp Lipid Metabolism Disorders/ 121062

45 "metabolic syndrome".mp. or exp Metabolic Syndrome/ 64458

46 exp Glucose Metabolism Disorders/ or "glucose metabolism disorder*".mp. 501684

47 "chronic kidney disease".mp. or exp Renal Insufficiency, Chronic/ 158459

48 "chronic kidney failure".mp. or exp Kidney Failure, Chronic/ 98837

49 exp Glomerular Filtration Rate/ or "reduced eGFR".mp. 48648

50 albuminuria.mp. or exp Albuminuria/ 22184

51 14 or 15 or 16 or 17 or 18 or 19 or 20 or 21 or 22 or 23 or 24 or 25 or 26 or 27 or 28 or 29 or 30 or 31 or 32 or 33 or 34 or 35 or 36 or 37 or 38 or 39 or 40 or 41 or 42 or 43 or 44 or 45 or 46 or 47 or 48 or 49 or 50 2620429

52 exp Epidemiologic Studies/ or "epidemiologic* stud*".mp. 2965608

53 exp Cohort Studies/ or "cohort stud*".mp. 2386823

54 (cohort* adj (study or studies)).mp. 477856

55 (cohort adj (analysis or analyses)).mp. [mp=title, abstract, original title, name of substance word, subject heading word, floating sub-heading word, keyword heading word, organism supplementary concept word, protocol supplementary concept word, rare disease supplementary concept word, unique identifier, synonyms] 10573

56 (follow up adj (study or studies)).mp. [mp=title, abstract, original title, name of substance word, subject heading word, floating sub-heading word, keyword heading word, organism supplementary concept word, protocol supplementary concept word, rare disease supplementary concept word, unique identifier, synonyms] 706643

57 (observational adj (study or studies)).mp. [mp=title, abstract, original title, name of substance word, subject heading word, floating sub-heading word, keyword heading word, organism supplementary concept word, protocol supplementary concept word, rare disease supplementary concept word, unique identifier, synonyms] 218811

58 (longitudinal observation* adj (study or studies)).mp. [mp=title, abstract, original title, name of substance word, subject heading word, floating sub-heading word, keyword heading word, organism supplementary concept word, protocol supplementary concept word, rare disease supplementary concept word, unique identifier, synonyms] 2680

59 exp Longitudinal Studies/ or longitudinal.mp. 351234

60 exp Retrospective Studies/ or "retrospective stud*".mp. 1057994

61 "randomised controlled trial".mp. 28078

62 RCT.mp. 29109

63 "controlled clinic* trial*".mp. 129336

64 (drug adj therapy).mp. [mp=title, abstract, original title, name of substance word, subject heading word, floating sub-heading word, keyword heading word, organism supplementary concept word, protocol supplementary concept word, rare disease supplementary concept word, unique identifier, synonyms] 2538212

65 human*.mp. 20948431

66 52 or 53 or 54 or 55 or 56 or 57 or 58 or 59 or 60 or 61 or 62 or 63 or 64 or 65 21435760

67 exp "Aged, 80 and over"/ or exp Aged/ 1527895

68 (aged or ageing or aging or elder* or geriatric* or gerontolog*).mp. [mp=title, abstract, original title, name of substance word, subject heading word, floating sub-heading word, keyword heading word, organism supplementary concept word, protocol supplementary concept word, rare disease supplementary concept word, unique identifier, synonyms] 6098338

69 ("advanced year*" or "advancing year*").mp. [mp=title, abstract, original title, name of substance word, subject heading word, floating sub-heading word, keyword heading word, organism supplementary concept word, protocol supplementary concept word, rare disease supplementary concept word, unique identifier, synonyms] 277

70 (old* adj (adult* or female* or male* or men or women or people or person)).mp. [mp=title, abstract, original title, name of substance word, subject heading word, floating sub-heading word, keyword heading word, organism supplementary concept word, protocol supplementary concept word, rare disease supplementary concept word, unique identifier, synonyms] 340987

71 "over 65".mp. 8805

72 68 or 69 or 70 or 71 6238902

73 "residential ho*".mp. 1432

74 "care ho*".mp. 44288

75 exp Homes for the Aged/ or exp Nursing Homes/ or "nursing ho*".mp. or exp Long-Term Care/ 81678

76 "long term care".mp. 41420

77 "care facilit*".mp. 54092

78 73 or 74 or 75 or 76 or 77 175799

79 13 and 51 and 66 and 72 and 78 1630

**Figure S1: 1.2 Web of Science**

ALL=(deprescribing OR polypharmacy OR inappropriate prescribing OR deintensif* OR Deprescrib* OR polymedication ) 17,645

ALL=(aged OR ageing OR aging OR geriatric OR gerontolog* OR advanced year* OR advancing year* ) 4,244,626

ALL=(diabetes mellitus type (2 OR two OR ii) OR “non insulin dependent diabetes” OR NIDDM OR T2DM OR DM2 OR “cardiovascular disease” OR “cardiometabolic disease”) 372,702

ALL=(stroke OR hypertension OR “high blood pressure” OR hyperlipid*emia OR dyslipid*emia OR “chronic kidney disease” OR CKD) 1,320,938

ALL=(epidemiological stud* OR follow up stud* OR observational stud* OR longitudinal stud* OR longitudinal observational stud* OR retrospective stud* OR randomised controlled trial* OR RCT OR controlled clinic* trial* OR drug therapy) 2,890,119

ALL=("nursing home" OR "care home*" OR "residential home") 38,916

#4 OR #3

#1 AND #2 AND #7 AND #5 AND #6

**Figure S1: 1.3 EMBASE**

Embase <1974 to 2022 March 22>

1 deprescription.mp. or exp Deprescriptions/ 876

2 deprescrib*.mp. 1810

3 deprescrip*.mp. 903

4 exp Drug Tapering/ 83920

5 (reduced or reduces or reducing or reduction).mp. 4481136

6 (ceased or ceasing or cessation*).mp. 159079

7 discontinu*.mp. 234636

8 stop*.mp. 231515

9 withdraw*.mp. 414141

10 taper*.mp. 37428

11 deintensification*.mp. 277

12 (Deprescri* or "de-prescri" or "unprescri*" or "cease" or "ceasing" or "withdraw" or "discontinu" or "stop" or "intermittent").mp. 259441

13 1 or 2 or 3 or 4 or 5 or 6 or 7 or 8 or 9 or 10 or 11 or 12 5323346

14 exp Diabetes Mellitus/ or exp Diabetes Mellitus, Type 2/ 1085493

15 "non-insulin dependent diabetes".mp. 296649

16 NIDDM.mp. 8306

17 T2DM.mp. 46136

18 DM2.mp. 4664

19 "type 2 diabete*".mp. 228352

20 "type ii diabete*".mp. 15080

21 "type two diabete*".mp. 459

22 (type 2 adj3 diabet*).mp. 247668

23 (type two adj3 diabet*).mp. 553

24 (type ii adj3 diabet*).mp. 19318

25 exp Cardiovascular Diseases/dt, ep, tu [Drug Therapy, Epidemiology, Therapeutic Use] 694032

26 cardiometabolic.mp. 25825

27 exp Hypertension/ or hypertension.mp. 1065002

28 exp Hemorrhagic Stroke/ or exp Stroke/ or exp Embolic Stroke/ or stroke.mp. or exp Thrombotic Stroke/ or exp Ischemic Stroke/ 677273

29 "cerebrovascular accident*".mp. 248867

30 exp Ischemic Attack, Transient/ or "transient isch*emic attack*".mp. 46805

31 "heart failure".mp. or exp Heart Failure/ 623367

32 exp Coronary Artery Disease/ or exp Myocardial Infarction/ or "coronary artery disease*".mp. or exp Myocardial Ischemia/ or exp Coronary Disease/ 753584

33 exp Peripheral Arterial Disease/ or exp Peripheral Vascular Diseases/ or "peripheral artery disease*".mp. 1982141

34 exp Arterial Occlusive Diseases/ or "arterial disease*".mp. 195006

35 ("arteriosclerosis" or "atherosclerosis").mp. 289330

36 exp Atherosclerosis/ or exp Arteriosclerosis/ or exp Plaque, Atherosclerotic/ or Atherosclerotic.mp. 293829

37 "heart attack".mp. 7214

38 ("NSTEMI" or "STEMI" or "ACS").mp. 80907

39 "acute coronary syndrome".mp. or exp Acute Coronary Syndrome/ 77152

40 exp Angina, Unstable/ or exp Angina Pectoris/ or angina.mp. 122929

41 (angina adj3 (stable or unstable)).mp. 46127

42 exp Dyslipidemias/ or dyslipid*emia.mp. or exp Hyperlipidemias/ 261303

43 exp Hypercholesterolemia/ or hypercholesterol*emia.mp. 86163

44 "lipid metabolism disorder*".mp. or exp Lipid Metabolism Disorders/ 399358

45 "metabolic syndrome".mp. or exp Metabolic Syndrome/ 113435

46 exp Glucose Metabolism Disorders/ or "glucose metabolism disorder*".mp. 1320211

47 "chronic kidney disease".mp. or exp Renal Insufficiency, Chronic/ 191870

48 "chronic kidney failure".mp. or exp Kidney Failure, Chronic/ 124285

49 exp Glomerular Filtration Rate/ or "reduced eGFR".mp. 126334

50 albuminuria.mp. or exp Albuminuria/ 40388

51 14 or 15 or 16 or 17 or 18 or 19 or 20 or 21 or 22 or 23 or 24 or 25 or 26 or 27 or 28 or 29 or 30 or 31 or 32 or 33 or 34 or 35 or 36 or 37 or 38 or 39 or 40 or 41 or 42 or 43 or 44 or 45 or 46 or 47 or 48 or 49 or 50 4717792

52 exp Epidemiologic Studies/ or "epidemiologic* stud*".mp. 4045491

53 exp Cohort Studies/ or "cohort stud*".mp. 902438

54 (cohort* adj (study or studies)).mp. 392971

55 (cohort adj (analysis or analyses)).mp. 823328

56 (follow up adj (study or studies)).mp. 72470

57 (observational adj (study or studies)).mp. 327371

58 (longitudinal observation* adj (study or studies)).mp. 4020

59 exp Longitudinal Studies/ or longitudinal.mp. 435871

60 exp Retrospective Studies/ or "retrospective stud*".mp. 1267771

61 "randomised controlled trial".mp. 37804

62 RCT.mp. 48537

63 "controlled clinic* trial*".mp. 504701

64 (drug adj therapy).mp. 4951075

65 human*.mp. 24365891

66 52 or 53 or 54 or 55 or 56 or 57 or 58 or 59 or 60 or 61 or 62 or 63 or 64 9820242

67 exp "Aged, 80 and over"/ or exp Aged/ 3334332

68 (aged or ageing or aging or elder* or geriatric* or gerontolog*).mp. 5510286

69 ("advanced year*" or "advancing year*").mp. 356

70 (old* adj (adult* or female* or male* or men or women or people or person)).mp. 503730

71 "over 65".mp. 14651

72 67 or 68 or 69 or 70 or 71 5740977

73 "residential ho*".mp. 8696

74 "care ho*".mp. 67537

75 exp Homes for the Aged/ or exp Nursing Homes/ or "nursing ho*".mp. or exp Long-Term Care/ 2109885

76 "long term care".mp. 149651

77 "care facilit*".mp. 106147

78 73 or 74 or 75 or 76 or 77 2263807

79 13 and 51 and 65 and 66 and 72 and 78 49768

80 limit 79 to (full text and human and yr="2000 -Current") 5162

**Figure S1: 1.4 Cochrane Library**

deprescribing OR polypharmacy OR inappropriate prescribing OR deintensif* OR Deprescrib* OR polymedication in Title Abstract Keyword

AND aged OR ageing OR aging OR geriatric OR gerontolog* OR advanced year* OR advancing year* )

AND ( diabetes mellitus type (2 OR ii OR two) OR “non insulin dependent diabetes” OR NIDDM OR T2DM or DM2 OR cardiovascular disease* OR cardiometabolic disease OR stroke OR hypertension OR high blood pressure OR apoplexy OR stroke OR TIA OR transient isch*emic attack OR CVA OR “cerebrovascular accident” OR “coronary artery disease” OR “coronary arterial disease” OR “peripheral vascular disease” OR PVA OR “heart failure” OR HF OR “heart attack” OR hyperlipid*emia OR dyslipid*emia OR “glucose metabolism disorder*” OR “non alcoholic fatty liver disease” OR NAFLD OR CKD OR “chronic kidney disease” OR “chronic renal disease” OR albuminuria in Title Abstract Keyword

AND epidemiological stud* OR follow up stud* OR observational stud* OR longitudinal stud* OR longitudinal observational stud* OR retrospective stud* OR randomised controlled trial* OR RCT OR controlled clinic* trial* OR drug therapy in Title Abstract Keyword - (Word variations have been searched)

**Figure S1: 1.5 Clinical Trials**

deprescribing OR polypharmac* OR inappropriate prescribing OR deintensif* OR Deprescrib* OR polymedication* OR withdraw* OR ceas* OR cessation OR discontinu* OR reduc* OR withheld OR substitut* OR stop* or eliminat* OR taper*

Diabetes Mellitus OR T2DM OR Type 2 Diabetes OR DMT2 OR DM OR Cardiovascular OR Hypertension OR Hyperlipidaemia OR CKD OR Chronic Kidney Disease OR Metabolic Syndrome OR Myocardial Infarction OR NSTEMI OR STEMI OR Angina OR Stroke

Study Type: All studies

Study Results: All studies

Age: Over 65+

Sex: All

619 Studies found for: deprescribing OR polypharmac* OR inappropriate prescribing OR deintensif* OR Deprescrib* OR polymedication* OR withdraw* OR ceas* OR cessation OR discontinu* OR reduc* OR withheld OR substitut* OR stop* or eliminat* OR taper* | Diabetes Mellitus OR T2DM OR Type 2 Diabetes OR DMT2 OR DM OR Cardiovascular OR Hypertension OR Hyperlipidaemia OR CKD OR Chronic Kidney Disease OR Metabolic Syndrome OR Myocardial Infarction OR NSTEMI OR STEMI OR Angina OR Stroke

## **Table S3: Eligibility Criteria**

| **Databases** | Medline  Cochrane databases and Cochrane central register of clinical trials  Clinicaltrials.gov.uk  EMBASE  CINAHL  Web of Science  Scopus |
| --- | --- |
| **Exposure of interest** | Type 2 diabetes  Cardiometabolic conditions – CVD (MI, angina [stable and unstable], stroke [incl. TIA], hypertension), CKD, diabetes, insulin resistance, non-alcoholic fatty liver disease, metabolic syndrome  Multi-morbidity  Aged >65 years old  End-of-life care, palliation, or best supportive care  Long term care facilities  Medication deprescribing – deprescribing of a minimum of 1 medication – stopping at least 1 medication, reducing the medication dose of at least 1 medication, tapering of a least 1 medication, or using an alternative medication for at least 1 regularly used medication  EXCLUDING – patients aged less than 65 years old, patients who do not have type 2 diabetes and/or other cardiometabolic conditions (type 1 diabetes is excluded), patients who are not on EOL care/palliation/best supportive care |
| **Geographic location of study** | Papers published in English  Global |
| **Language** | English language papers  Unable to translate – due to university constraints |
| **Participants** | Elderly patients >=65 years old  End of life care  If data can be extracted from the paper for patients >= 65 years old it can be included  Participants or a separately reported subgroup with T2DM and/or other cardiometabolic conditions  Deprescribing interventions discussed  Reported outcomes discussed  Control group – same demographics |
| **Peer Review** | Peer reviewed articles will be included  Articles that are not peer reviewed will also be included |
| **Reported outcomes** | Outcomes must be reported  Morbidity and mortality  Complications – DKA, HHS, Charcot foot, ulcers, infections, hypo-/hyper-glycaemia, retinopathy, peripheral neuropathy (diabetic neuropathy), MI, reduced renal function, changes to insulin sensitivity, strokes, PAD, thromboembolic events.  Hospitalisations (admission to hospital, visits to hospital – any secondary care interventions)/GP visits/GP home visits  Patient satisfaction  Quality of life  Falls/Syncope  Laboratory values – HbA1c marker  At least 1 reported outcome |
| **Setting** | End-of-life care/palliation/best supportive care  Nursing homes  Residential homes  Care homes  Long term care facilities  Community living centres |
| **Study design** | Observational studies  RCT’s  Non-randomised prospective studies  Prospective cohort studies  Retrospective cohort studies  Any randomised or non-randomised design  RCT or observational studies with comparison/control groups |
| **Type of publication** | Meta-analyses  Reviews of clinical trials – controlled trial registers  Evidence-based medicine  Consensus development conferences  Guidelines  Unpublished literature  Research not published in a journal  Full article or abstract publications  Any medical deprescribing intervention (as outlined above) for type 2 diabetes and/or cardiometabolic conditions  Abstracts that include one or more keywords  Peer reviewed journals |

## **Table S4: Study Quality Assessments using Newcastle Ottowa Scale for cohort studies**

|  | Selection | | | | Comparability | Outcome | | |  |
| --- | --- | --- | --- | --- | --- | --- | --- | --- | --- |
| Lead Author | **Representative of the exposed cohort** | **Selection of the non-exposed cohort** | **Ascertainment of exposure** | **Demonstration that outcome of interest was not present at start of study** | **Comparability of cohorts on the basis of the design/analysis** | **Assessment of outcome** | **Was follow up long enough for outcomes to occur** | **Adequacy of follow up cohorts** | **Score out of 9** |
| Al Aqqad, 2014 |  | ★ | ★ | ★ | ★ | ★ | ★ |  | 6 |
| Niznik, 2020 | ★ |  |  | ★ | ★ | ★ | ★ |  | 5 |
| Onder, 2019 | ★ |  | ★ | ★ | ★ |  | ★ | ★ | 5 |
| Sjoblem, 2008 |  | ★ | ★ | ★ | ★ | ★ | ★ |  | 5 |
| Song, 2018 | ★ | ★ | ★ | ★ | ★ | ★ |  | ★ | 7 |
| Springer, 2020 | ★ | ★ |  | ★ | ★ | ★ | ★ |  | 6 |
| Thorpe, 2020 | ★ |  |  | ★ | ★ |  | ★ | ★ | 5 |
| Vu, 2021 | ★ |  |  | ★ | ★ | ★ |  | ★ | 5 |
| Wilkins, 1985 |  | ★ | ★ |  | ★ | ★ | ★ |  | 5 |

## **Table S5: Study Quality Assessments using Newcastle Ottowa Scale for RCTs**

|  | Selection | | | | Comparability | Outcome | | |  |
| --- | --- | --- | --- | --- | --- | --- | --- | --- | --- |
| Lead Author | **Is the case definition adequate?** | **Representativeness of the cases** | **Selection of controls** | **Definition of controls** | **Comparability of cases and controls on the basis of design or analysis** | **Ascertainment of exposure** | **Same method of ascertainment for cases and controls** | **Non-response rate** | **Score out of 9** |
| Gulla, 2018 | ★ |  | ★ | ★ | ★ | ★ | ★ |  | 6 |
| Kutner, 2015 | ★ |  | ★ | ★ | ★ | ★ | ★ |  | 6 |
| Myers, 1982 | ★ |  | ★ |  | ★ | ★ | ★ |  | 5 |

## **Table S6: Study Quality Assessments using NIH Quality Assessment Tool for pre post studies**

| Lead Author | Question 1 | Question 2 | Question 3 | Question 4 | Question 5 | Question 6 | Question 7 | Question 8 | Question 9 | Question 10 | Question 11 | Question 12 | Score out of 12 |
| --- | --- | --- | --- | --- | --- | --- | --- | --- | --- | --- | --- | --- | --- |
| Forman, 1991 | ★ | ★ | ★ |  |  | ★ | ★ |  | ★ |  | ★ |  | **7** |
